# *RUNX1* Alterations in Pediatric Myeloid Malignancies: Divergent Germline and Somatic Trajectories

**DOI:** 10.3390/ijms27114805

**Published:** 2026-05-26

**Authors:** Ana Maria Bicǎ, Andra Daniela Marcu, Cristina Georgiana Jercan, Letiția Elena Radu, Irina Avramescu, Cerasela Jardan, Dumitru Jardan, Onda Tabita Cǎlugǎru, Cristina Mambet, Anca Colițǎ

**Affiliations:** 1 Faculty of Medicine, University of Medicine and Pharmacy Carol Davila, 050474 Bucharest, Romania; ana-maria.birsan@drd.umfcd.ro (A.M.B.); cristina.jercan@umfcd.ro (C.G.J.); letitia.radu@umfcd.ro (L.E.R.); irina.avramescu@drd.umfcd.ro (I.A.); cerasela.jardan@umfcd.ro (C.J.); onda-tabita.lupu@drd.umfcd.ro (O.T.C.); cristina.mambet@umfcd.ro (C.M.); anca.colita@umfcd.ro (A.C.); 2 Department of Pediatrics, and Bone Marrow Transplantation Unit, Fundeni Clinical Institute, 022328 Bucharest, Romania; 3 Department of Hematology Laboratory, Fundeni Clinical Institute, 022328 Bucharest, Romania; 4 Molecular Biology Laboratory, MedLife, 010719 Bucharest, Romania; djardan@medlife.ro; 5 Department of Hematology, Emergency University Hospital Bucharest, 050098 Bucharest, Romania

**Keywords:** *RUNX1*, germline predisposition, pediatric myelodysplastic syndrome, acute myeloid leukemia, mixed-phenotype acute leukemia, hematopoietic stem cell transplantation, monosomy 7, next-generation sequencing

## Abstract

*RUNX1* alterations contribute to pediatric myeloid malignancies through both germline predisposition syndromes and somatic leukemogenic events, but their clinical and biological significance in children remains incompletely defined. This retrospective single-center study evaluated six pediatric patients with myelodysplastic syndromes or acute leukemias harboring *RUNX1* variants, integrating clinical, cytogenetic, and targeted next-generation sequencing data, with germline status confirmed using non-hematopoietic tissues. Three patients carried germline *RUNX1* variants, characterized by antecedent cytopenias, dysplastic features, and increased treatment-related toxicity, including severe infections, persistent cytopenias, and transplant-related mortality. In contrast, somatic *RUNX1* alterations were associated with overt high-risk disease, frequently accompanied by complex cytogenetics or monosomy 7, and demonstrated heterogeneous outcomes ranging from sustained remission to post-transplant relapse. Mixed-phenotype acute leukemia was observed in both groups. These findings support a model of *RUNX1*-driven leukemogenesis, in which germline and somatic alterations represent distinct yet interconnected trajectories, while highlighting the importance of distinguishing variant origin for risk stratification, donor selection, and therapeutic decision-making in pediatric myeloid malignancies. Given the small cohort size, the findings remain descriptive and require validation in larger prospective studies.

## 1. Introduction

Myelodysplastic syndromes (MDSs) represent a heterogeneous group of clonal hematopoietic disorders characterized by ineffective hematopoiesis, persistent cytopenias, and a variable risk of progression to acute myeloid leukemia (AML) [1,2,3]. Although MDS/AML predominantly affects older adults, early-onset disease in children frequently reflects an underlying germline predisposition rather than the age-related accumulation of somatic mutations typical of adult cases [1,2].

Advances in genomic sequencing have expanded the recognition of inherited susceptibility to myeloid malignancies, including both classical bone marrow failure syndromes and more recently defined entities such as *GATA2* deficiency, *SAMD9/SAMD9L*-associated disorders, and *RUNX1*-related familial platelet disorder [4,5,6,7,8,9,10,11,12,13,14]. These conditions often present subtle or nonspecific features and may lack a clear family history, contributing to underdiagnosis [11,15]. Among these, *RUNX1* plays a central role as a master transcriptional regulator of hematopoiesis, governing stem cell differentiation and lineage commitment through the core-binding factor (CBF) complex, in which it functions as the DNA-binding subunit interacting with CBFB to regulate gene expression programs essential for hematopoietic and immune development [16,17,18]. Germline *RUNX1* variants cause familial platelet disorder with predisposition to myeloid malignancies, whereas somatic alterations are recurrently observed in MDS/AML [19,20,21,22]. Although *RUNX1* mutations are relatively common and associated with adverse outcomes in adult AML/MDS, they are rare in the pediatric setting (~2%) and their clinical significance remains incompletely defined [23,24]. Failure to identify these conditions may result in inappropriate donor selection, lack of optimal treatment strategies, increased transplant-related toxicity, and missed opportunities for genetic counseling and surveillance [4,25].

In this retrospective single-center cohort study, we comparatively evaluated pediatric patients with germline and somatic *RUNX1* alterations, focusing on their clinical presentation, molecular landscape, treatment course, and transplant-related outcomes. By integrating genomic and clinical data across a uniformly evaluated cohort, we sought to define biologically meaningful patterns associated with *RUNX1*-driven pediatric myeloid malignancies and to explore the relationship between *RUNX1* context and lineage ambiguity, particularly mixed-phenotype acute leukemia (MPAL). Our findings underscore the importance of integrating genomic context into clinical decision-making in pediatric hematologic malignancies.

## 2. Results

### 2.1. Cohort Characteristics

This series includes six pediatric cases with *RUNX1*-altered hematologic malignancies, equally divided between germline and somatic lesions. Age at diagnosis ranged from 4 to 17 years (median, 7.5 years), with a male-to-female ratio of 2:1. The clinical spectrum comprised two MDS-spectrum disorders and four acute leukemias, with five of six patients undergoing allogeneic hematopoietic stem cell transplantation (HSCT). Detailed clinical, molecular, and outcome data are summarized in Table 1 and Table 2.

None of the patients had a documented family history suggestive of hereditary hematologic disease. Nevertheless, three cases were confirmed to harbor germline *RUNX1* variants. Notably, targeted familial testing did not identify the variant in first-degree relatives, supporting the occurrence of apparently sporadic constitutional *RUNX1* predisposition in the pediatric setting.

From a clinical perspective, distinguishing germline from somatic *RUNX1* alterations proved highly relevant. Germline cases were characterized by antecedent cytopenic or dysplastic features and substantial treatment-related morbidity, including prolonged cytopenias, severe infections, one pre-transplant non-relapse death, and two transplant-related deaths following grade IV acute graft-versus-host disease (GvHD) after matched unrelated donor (MUD) HSCT. In contrast, somatic *RUNX1* lesions occurred in overt high-risk disease, frequently associated with complex cytogenetics or monosomy 7, and showed heterogeneous outcomes ranging from durable remission to post-transplant relapse.

**Figure 1:**
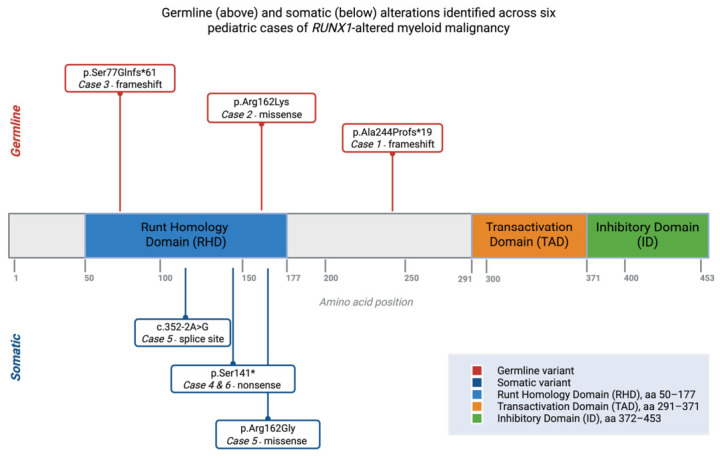
RUNX1 protein domains and distribution of identified germline/somatic variants (Created in BioRender. Ana, A. (2026) https://BioRender.com/twxt1jd).

The functional consequences of the identified variants are further illustrated by their distribution across the *RUNX1* protein (Figure 1), with germline alterations clustering within or upstream of the Runt homology domain and somatic lesions predominantly affecting known mutational hotspots within the same region. Consistent with this pattern, variants affecting codon R162 localize to a critical DNA-binding hotspot, while splice-site alterations involving canonical acceptor sites are predicted to disrupt RNA processing and result in loss of function (Table 2). In cases with complex genomic backgrounds, such as monosomy 7 or multiple co-occurring mutations, *RUNX1* alterations are best interpreted within a cooperative leukemogenic framework, acting as progression-associated events rather than isolated drivers.

### 2.2. Case 1

A 4-year-old girl presented with recurrent fever and cytopenias, including anemia (Hb 8.8 g/dL) and thrombocytopenia (75 × 10^9^/L), associated with peripheral blood monocytosis. Blood smear demonstrated dysplastic features, with Pelger–Huët-like neutrophils and erythrocytes with basophilic stippling. Bone marrow (BM) examination further revealed left-shifted myelopoiesis, with 2% blasts and dysplastic megakaryopoiesis, consistent with a myelodysplastic/myeloproliferative neoplasm. Targeted next-generation sequencing (NGS) identified a *RUNX1* c.723_729dup (p.A244Pfs*19) variant with an allele frequency (VAF) of 43%. Analysis of DNA from buccal swab, hair follicles and skin biopsy confirmed the variant as germline. As reported in ClinVar, this germline variant is associated with hereditary thrombocytopenia and hematologic cancer predisposition syndrome, and the ClinGen Myeloid Malignancy Expert Panel Specifications of the ACMG/AMP Variant Interpretation Guidelines Version 2 assign it as pathogenic, leading to loss of the transactivation domain (TAD) [20].

The patient received six cycles of AZA for progressive marrow involvement, and achieved hematologic response, followed by MUD 10/10 HSCT with thiotepa–treosulfan–fludarabine (TT-Teo-Flu) conditioning and ciclosporine (CSA), methotrexate (MTX) and anti-thymocyte globulin (ATG) for GvHD prophylaxis. Despite successful engraftment with full donor chimerism, the post-transplant course was complicated by refractory acute gastrointestinal and cutaneous GvHD and cytomegalovirus reactivation, ultimately leading to fatal complications on day +80 post-transplant.

### 2.3. Case 2

A 15-year-old girl with a history of chronic mild thrombocytopenia since early childhood was evaluated for erythematous maculopapular rash, severe headache, and marked leukocytosis. Laboratory evaluation revealed leukocytosis (141 × 10^9^/L), normocytic anemia (Hb 10.8 g/dL), thrombocytopenia (31 × 10^9^/L), and 78% blasts. BM examination confirmed MPAL diagnosis. Targeted NGS identified a *RUNX1* p.R162K (c.485G>A) variant (VAF 49.1%), subsequently confirmed as germline. This variant is classified as “likely pathogenic” according to ACMG guidelines and as reported in ClinVar in association with hereditary thrombocytopenia and hematologic cancer predisposition syndrome. The affected residue, codon 162, is located within the highly conserved Runt homology domain (RHD), the DNA-binding region of RUNX1 protein [26].

The patient received induction chemotherapy followed by intensification treatment including sorafenib following detection of *FLT3-ITD* and azacitidine/venetoclax (AZA/VEN), achieving 0.2% measurable residual disease (MRD) prior to transplantation. She underwent MUD 9/10 HSCT with busulfan–cyclophosphamide–melphalan (BuCyMel) conditioning and post-transplant cyclophosphamide (PTCy) for GvHD prophylaxis. Although full donor chimerism was achieved, the post-transplant course was complicated by severe acute GvHD involving skin, eye and gastrointestinal tract, recurrent pulmonary infections, and multiorgan complications, ultimately resulting in death four months after transplantation.

### 2.4. Case 3

Clinical assessment identified fever, extensive ecchymoses, and hepatosplenomegaly in a 9-year-old boy. Laboratory evaluation revealed marked hyperleukocytosis (WBC 146 × 10^9^/L) with neutrophilia and monocytosis, severe normocytic anemia (Hb 4 g/dL), and profound thrombocytopenia (9 × 10^9^/L). BM examination demonstrated MPAL and myelodysplastic features. Cytogenetic analysis revealed complex karyotype (47,XY,+21[7]/44~47,XY,+21,+r,+mar,inc[cp3]). Targeted NGS identified a *RUNX1* exon 4 duplication, c.227dupG (p.Ser77Glnfs*61), further confirmed as a germline variant that, according to ClinVar, has been reported in hereditary thrombocytopenia and hematological cancer predisposition syndrome associated with *RUNX1* and is assigned as pathogenic. The frameshift duplication introduces a premature stop codon and results in truncation of the RUNX1 protein after amino acid 77 of the normal 453 residues, leading to loss of the major functional domains of the protein and predicted loss of function. Additional somatic variants (germline testing negative) were identified: *NRAS* (c.37G>C, p.G13R, VAF 46.3%) and *CBL* (c.1096-1G>T, VAF 38.9%) classified as Tier I, pathogenic according to AMP/ACMG guidelines. A *STAT3* mutation (c.1981G>T, p.D661Y, VAF 16.8%) was classified in Tier II with potential clinical significance.

The patient achieved an initial response following chemotherapy, with a post-induction MRD of 0.02%. However, the clinical course during treatment was characterized by prolonged cytopenias, marked susceptibility to severe infections (recurrent pneumonias and sepsis), difficult tolerance to chemotherapy, and prolonged transfusion dependency. Bridging therapy with AZA/VEN resulted in temporary disease control. At the time of evaluation for allo-HSCT, no matched unrelated donor was available; a fully matched sibling donor was identified but raised concerns about a possible underlying constitutional syndrome; therefore, the family was tested for *RUNX1* germline (negative results). Overt disease progression was documented with 12% blasts on FCM, prompting reinitiation of intensive chemotherapy. The patient’s course was ultimately complicated by septic shock from extensively drug-resistant Klebsiella, leading to death before transplantation.

### 2.5. Case 4

A 17-year-old male presented with severe cytopenias, including anemia (Hb 6.2 g/dL), leukopenia (1.94 × 10^9^/L) with marked neutropenia (0.45 × 10^9^/L), and profound thrombocytopenia (11 × 10^9^/L). He reported bilateral knee arthralgia associated with functional impairment of the lower limbs. BM evaluation established MPAL diagnosis. Cytogenetic analysis revealed a complex karyotype with chromosomal instability. Targeted NGS identified a *RUNX1* nonsense variant, p.Ser141* (COSV55867703) with a VAF of 41.9%, classified as Tier I, pathogenic (AMP, ACMG). This variant introduces a premature stop codon resulting in truncation of the RUNX1 protein and likely loss of function. Additional mutations were detected in *JAK3*, *MDM4*, *MYCL*, *NOTCH1*, *SMC1A*, *ATM*, and *FANCA*, together with a RUNX1::AFF3 fusion. Following induction therapy, the patient achieved MRD negativity.

The patient underwent MSD allo-HSCT using FluThioBu and CSA plus MTX for GvHD prophylaxis. Neutrophil engraftment occurred on day +18. The post-transplant course was uneventful, with no GvHD or major complications. The patient remains alive and in complete remission (CR) 30 months after transplantation.

### 2.6. Case 5

A 6-year-old boy presented with a five-week history of pallor, fatigue, weight loss, high fever, and precordial pain. Laboratory evaluation revealed severe pancytopenia with profound anemia (Hb 3.5 g/dL), leukopenia (2.86 × 10^9^/L) with neutropenia (0.29 × 10^9^/L), and thrombocytopenia (35 × 10^9^/L). BM examination established the diagnosis of acute megakaryoblastic leukemia (AML-M7). Cytogenetic analysis demonstrated monosomy 7, while targeted NGS identified two *RUNX1* variants, classified as Tier I, likely pathogenic according to ACMG/AMP criteria, consistent with somatic alterations contributing to leukemogenesis. The first variant, *RUNX1* c.484A>G, p.Arg162Gly (COSV55867213), with a VAF of 9.1% results in the substitution of arginine with glycine at codon 162, a residue located within the DNA-binding RHD that is known to directly interact with DNA [27,28,29,30]. The position represents a recognized mutational hotspot in myeloid malignancies [31,32]. The variant is absent from population databases, including gnomAD v2 and v3, and has been previously reported as a somatic alteration in cases of MDS/AML. The second variant, *RUNX1* c.352-2A>G, p.unknown, (COSV55875071) with a VAF of 18.4%, affects the canonical splice acceptor site and is predicted to disrupt normal mRNA splicing. This splice-site alteration has been reported as a somatic variant in cancer, although data in hematologic malignancies remain limited. The predicted loss of normal splicing consists of a deleterious effect on *RUNX1* function.

The patient achieved CR after induction therapy. At the parents’ request, further treatment was continued abroad, where he received allogeneic HSCT. Detailed transplant-related data were unavailable; the patient ultimately died from disease relapse following transplantation.

### 2.7. Case 6

A 5-year-old boy, a refugee from the Gaza region, presented with a two-month history of pallor, ecchymoses, and lymphadenopathy. Initial laboratory evaluation revealed pancytopenia, and bone marrow examination performed prior to referral showed >50% blasts. At admission to our center, the patient exhibited pallor, cutaneous hemorrhagic lesions, and mild cervical lymphadenopathy without hepatosplenomegaly. Laboratory tests demonstrated leukocytes 9.7 × 10^9^/L, severe neutropenia (ANC 0.54 × 10^9^/L), normocytic anemia (Hb 10.5 g/dL), and profound thrombocytopenia (16 × 10^9^/L). BM evaluation revealed hypocellularity with approximately 18% myeloid blasts, consistent with MDS with excess blasts (MDS-EB). Cytogenetic analysis showed monosomy 7, confirmed by FISH (7q deletion in 65% of nuclei).

Targeted NGS identified a truncating *RUNX1* variant, c.422C>A (p.Ser141)*, with a VAF of approximately 24%. This nonsense alteration introduces a premature stop codon within the RHD, located upstream of the TAD of *RUNX1* [22], and is therefore predicted to result in truncation of the protein with partial/complete loss of key functional regions, consistent with a loss-of-function effect. Although the *RUNX1* p.S141 variant has not yet been functionally characterized, it has been previously reported in the COSMIC database (COSV55867703; accessed April 2021). Additional alterations were identified in *CUX1* and *BRCA2*, while a *SAMD9L* (VAF~29%) variant proved to be germline. Although currently classified as a variant of uncertain significance, its absence from population databases and its reported association with monosomy 7-associated MDS support a potential role in germline predisposition to MDS.

The patient initially received AZA therapy with stable disease and subsequently underwent MSD allo-HSCT (sibling tested germline-negative for the *SAMD9L* variant) after myeloablative conditioning (BuCy) with tacrolimus and MTX for GvHD prophylaxis. Early post-transplant evaluation revealed mixed donor chimerism, prompting withdrawal of immunosuppression and DLI, which resulted in restoration of full donor chimerism, mild GvHD and a favorable clinical outcome.

## 3. Discussion

*RUNX1* alterations in pediatric hematologic malignancies define two distinct biological contexts: germline predisposition and somatic leukemogenic events. Germline *RUNX1* variants confer a substantial risk of myeloid malignancies, with approximately 27% of patients developing AML and 13% MDS, and may underlie familial platelet disorder with associated myeloid malignancy (*RUNX1*-FPDMM), an autosomal dominant syndrome characterized by thrombocytopenia, platelet dysfunction, and progression through a preleukemic state requiring secondary hits for transformation [33,34,35,36,37]. However, the lack of overt clinical features or a negative family history does not exclude an underlying germline predisposition to MDS/AML. In the pediatric cohort reported by De Leon et al., most germline carriers presented in early childhood with persistent thrombocytopenia, additional cytopenias, and marrow dysplasia, often followed by a prolonged preleukemic phase before progression to MDS or AML [38]. Our germline cases shared similar baseline hematologic features but appeared to progress more rapidly to overt leukemia, likely reflecting both clinical heterogeneity and delayed recognition.

At the biological level, leukemogenesis in germline *RUNX1* deficiency follows a multistep clonal evolution model in which the inherited variant establishes a predisposing hematopoietic background, while secondary somatic events drive clonal expansion and malignant transformation. These include recurrent second hits affecting *RUNX1* itself, as well as cooperating alterations in genes such as *BCOR*, *SRSF2*, *TET2*, and *PHF6*, often accompanied by chromosome 21 abnormalities and, in some cases, signaling pathway activation [39,40,41,42]. In this context, clonal hematopoiesis emerges as a critical intermediate phase, frequently detectable in asymptomatic carriers and likely serving as a direct precursor to overt malignancy [43]. In contrast to sporadic AML, germline *RUNX1*-associated disease shows a relative paucity of adverse-risk features, including infrequent *ASXL1* mutations and monosomal karyotypes, supporting a distinct biological trajectory. Despite this, variant localization within *RUNX1* does not correlate with phenotype, and marked intra- and interfamilial heterogeneity is observed, consistent with a classical “two-hit” model requiring additional cooperating events for leukemic transformation [39,40].

This complementary framework is further illustrated by Case 2, which highlights both the biological complexity and the diagnostic pitfalls of germline disease. In this patient, a VAF of 49.1% was ultimately confirmed as germline in non-hematopoietic tissue, supported by a long-standing history of thrombocytopenia despite the absence of a recognized family history. This finding is consistent with the biology of *RUNX1*-FPDMM, where de novo occurrence, variable expressivity, or subtle familial phenotypes may obscure inheritance patterns. Leukemic progression may involve second-hit events affecting the wild-type allele or generating allelic imbalance—such as additional *RUNX1* lesions, trisomy 21, or copy-neutral loss of heterozygosity—thereby increasing the apparent VAF beyond 50% in leukemic samples. Accordingly, VAF should not be interpreted in isolation but integrated with clinical context and germline testing. The co-occurrence of *FLT3-ITD* in this case further supports a cooperative model of leukemogenesis and may have contributed to transformation within the inherited *RUNX1* background, particularly in the setting of lineage ambiguity such as MPAL [41,42].

In contrast, somatic *RUNX1* alterations occurred in overt high-risk disease, frequently associated with monosomy 7 or complex karyotype, findings that were also observed in our patients, and should be interpreted within the broader molecular and cytogenetic architecture rather than as an isolated biomarker. While in adults *RUNX1* mutations are linked to adverse-risk features and secondary-like AML biology [43], their prognostic impact in pediatric AML appears less uniform, remaining highly context-dependent and influenced by co-occurring cytogenetic abnormalities and cooperating molecular lesions [23,44]. Case 4 (somatic *RUNX1*-associated MPAL) exemplifies this paradigm, with *RUNX1* embedded within a complex genomic network involving JAK/STAT and *NOTCH* pathway alterations, supporting the concept that lineage ambiguity and aggressive disease biology arise from synergistic transcriptional and signaling dysregulation rather than a single defining event [45].

Beyond cooperating genetic alterations, increasing evidence suggests that *RUNX1* deficiency promotes leukemogenesis through epigenetic dysregulation. RUNX1 regulates hematopoietic differentiation through interactions with chromatin-remodeling complexes, including SWI/SNF components, and histone-modifying enzymes, such as HDAC1/2, Sin3A, and p300/CBP [16]. In core-binding factor leukemias, RUNX1 fusion proteins such as RUNX1::RUNX1T1 recruit transcriptional repressors, leading to promoter hypermethylation and the silencing of genes involved in differentiation and apoptosis [46,47,48]. Similarly, *RUNX1* disruption in AML has been associated with aberrant DNA methylation, altered chromatin accessibility, and repression of *RUNX1*-regulated targets, partly through impaired interaction with regulators such as *TET2* and *CEBPA* [49,50]. These alterations may promote stem-cell-like states, clonal persistence, and defective hematopoietic differentiation [51]. This concept appears particularly relevant in pediatric myeloid malignancies, where recurrent co-alterations frequently involve genes with epigenetic regulatory functions, including *ASXL1*, *EZH2*, *TET2*, *DNMT3A*, *BCOR*, and cohesion-complex members [52]. Additional mechanisms, such as disruption of long-range chromatin organization or imbalance between RUNX1 isoforms, may further contribute to disease evolution [53].

These processes may be especially relevant in MPAL, where altered regulation of lineage specification could contribute to phenotypic plasticity and lineage ambiguity. Single-cell multiomic studies have demonstrated enrichment of *RUNX1*-associated regulatory programs across heterogeneous MPAL blast populations, supporting a role for RUNX1-centered networks in aberrant lineage specification [54]. In this context, the epigenetic vulnerability associated with RUNX1 disruption may also provide a biological rationale for the activity of hypomethylating agents. Notably, several patients in our cohort showed clinical benefit from hypomethylating-based therapeutic approaches, although these observations remain descriptive and require validation in larger studies. Collectively, these observations suggest that *RUNX1*-associated leukemogenesis reflects dynamic interplay between genetic and epigenetic mechanisms during clonal evolution, contributing to impaired differentiation and mixed-phenotype leukemic states in pediatric disease [52].

This broader cooperative framework may also help explain biologically complex pediatric myeloid neoplasms arising in predisposition-associated contexts rather than through isolated leukemogenic events. In this regard, Case 6 was excluded from the somatic comparator framework, as its biological profile (pediatric MDS phenotype, monosomy 7, a germline *SAMD9L* VUS and *CUX1* lesions) more strongly supports predisposition-associated disease with secondary *RUNX1* evolution rather than a “pure” somatic *RUNX1*-associated neoplasm. This background is biologically important because functionally relevant germline *SAMD9/SAMD9L* variants can impose antiproliferative stress on hematopoietic progenitors, while hematopoietic escape through chromosome 7 loss may simultaneously alleviate this pressure and promote MDS evolution. In pediatric MDS, monosomy 7 is a major red flag for constitutional predisposition, most commonly involving *GATA2* or *SAMD9/SAMD9L*, and is frequently accompanied by secondary drivers such as *RUNX1*, *ASXL1*, *SETBP1*, or RAS-pathway lesions. Accordingly, in this case, the *RUNX1* alteration is more plausibly interpreted as a progression-associated event within a biologically complex, predisposition-like background than as an isolated defining lesion [13,42].

Although germline *RUNX1* variants are well established in myeloid malignancies and T-lineage leukemias (particularly early T-cell precursor (ETP) phenotypes), their association with pediatric MPAL remains poorly defined. Current evidence is mainly from somatic datasets, where *RUNX1* is among the most frequently mutated genes in ambiguous lineage leukemias, especially B/myeloid MPAL, and has been associated with clinical and biological features overlapping with *RUNX1*-mutated AML [55,56]. In contrast, germline *RUNX1* has not been systematically linked to MPAL. In our cohort, MPAL accounted for half of all *RUNX1*-altered cases, including two germline B/myeloid and one somatic T/myeloid presentation, suggesting a possible enrichment compared with the existing literature. This observation raises the possibility that germline *RUNX1* may, in some cases, predispose to impaired lineage commitment and ambiguous immunophenotypes, consistent with the known role of *RUNX1* in hematopoietic differentiation and lineage specification. However, given the limited cohort size and absence of a systematic comparison group, these findings remain exploratory and require validation in larger studies before implications for disease classification can be considered [57].

From a clinical perspective, distinguishing germline from somatic *RUNX1* status has direct therapeutic implications, as leukemic samples may obscure constitutional origin and require confirmation in non-hematopoietic tissue [58]. This distinction is critical for screening, donor selection, family counseling, and transplant strategy, given the association of unrecognized germline variants with graft-related complications and inferior outcomes [58,59]. While prior studies in mixed adult–pediatric cohorts suggest relatively favorable outcomes in germline *RUNX1*-associated malignancies [37], our findings indicate a more complex clinical course in pediatric patients, distinct from other predisposition syndromes such as GATA2 deficiency [10]. Notably, *RUNX1*-associated predisposition is typically characterized by a prolonged preleukemic latency, with a median age of malignancy onset in adulthood [37,40], which may partly account for the more favorable outcomes reported in these mixed study groups. In contrast, the early disease onset observed in our pediatric cases likely reflects a more aggressive biological trajectory and/or the presence of high-risk cooperating events. In our cohort, germline *RUNX1* cases were characterized less by primary chemoresistance than by substantial treatment- and transplant-related complications, including severe acute GvHD, recurrent infections, and early mortality after HSCT, as illustrated by Cases 1 and 2, while Case 3 further highlighted marked treatment intolerance with prolonged cytopenias and fatal infectious complications precluding transplantation.

Collectively, these observations suggest that in pediatric *RUNX1*-associated disease, host factors and treatment-related toxicity may play a disproportionate role in determining outcome, potentially attenuating the survival advantage reported in larger cohorts. These findings support the need for integrated genomic evaluation and risk-adapted therapeutic strategies, with particular emphasis on early recognition of germline predisposition and close monitoring for infectious and transplant-related complications [59,60].

**Figure 2:**
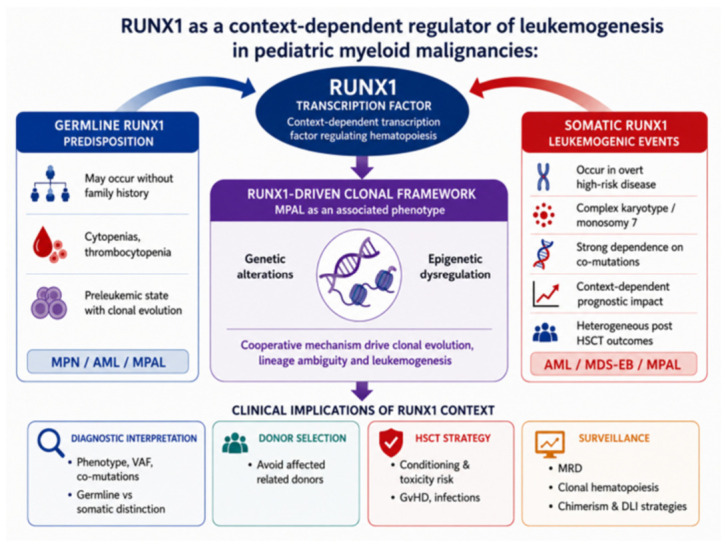
Germline and somatic *RUNX1* alterations in pediatric hematologic malignancies: biological trajectories and clinical implications (created in BioRender. Ana, A. (2026) https://BioRender.com/61k859m).

Overall, these observations may support a broader spectrum of *RUNX1*-associated leukemogenesis, in which distinct hematologic phenotypes arise through context-dependent patterns of clonal evolution. Based on this limited series, MPAL appears as a potentially associated phenotype that may reflect impaired lineage commitment; however, this interpretation remains descriptive and should be considered hypothesis-generating pending validation in larger cohorts (Figure 2).

This study has several important limitations. The cohort comprises only six cases, precluding statistical analysis and limiting interpretation of the observed associations. The absence of a systematic comparison cohort prevents inferential conclusions regarding clinical or prognostic differences associated with germline versus somatic *RUNX1* alterations. In addition, the retrospective single-center design introduces inherent selection and ascertainment biases, while incomplete transplant-related data for one case limited outcome assessment. Functional validation of *RUNX1* variants was not performed, and variant classification was based on published databases and computational pathogenicity prediction methods. Accordingly, the findings presented here are intended to contribute to the biological and clinical characterization of *RUNX1*-associated pediatric myeloid malignancies and to support future prospective investigations rather than establish prognostic or therapeutic recommendations.

## 4. Materials and Methods

### 4.1. Study Design and Patient Selection

This retrospective, single-center observational study was conducted at Fundeni Clinical Institute, Bucharest, Romania. This study included pediatric patients with myeloid malignancies in whom targeted next-generation sequencing (NGS) identified alterations in the *RUNX1* gene. Patients managed between January 2016 and December 2024 and aged 0–18 years at initial presentation were eligible if they had a diagnosis of MDS, AML, or mixed-phenotype acute leukemia (MPAL). Suspicion of an underlying germline predisposition was based on early age at onset, syndromic features, atypical disease course, and/or suggestive molecular findings. Given the limited cohort size, this study is designed as a descriptive, hypothesis-generating case series. No statistical comparisons between subgroups were performed or intended, and no prognostic inferences should be drawn from the reported outcomes.

### 4.2. Diagnostic and Molecular Evaluation

All patients underwent comprehensive diagnostic evaluation including complete blood count, peripheral blood smear, bone marrow morphology, immunophenotyping by multiparametric flow cytometry (FCM) used also for measurable residual disease (MRD), and cytogenetic analysis by G-banding karyotype and fluorescence in situ hybridization (FISH). Targeted DNA sequencing of patient samples was performed with TruSight Oncology 500 (Illumina, San Diego, CA, USA), including targeted sequencing of DNA from 523 genes and RNA from 1385 genes for a total panel size of 1.94 Mb (RNA panel was substituted with RNA pan cancer panel). Also, MSI and TMB measurement was performed. Sequenced genes were covered for the full exonic regions or exonic hot spots. According to the manufacturer’s guidelines, 500 ng of genomic DNA per sample was used to generate DNA libraries, and 500 ng of RNA was used to generate RNA libraries for fusion gene detection. Targeted sequencing was run on the NextSeq 550Dx instrument. For the secondary analysis, sequencing data was analyzed using DRAGEN TSO500 v2.2 software (Illumina, San Diego, CA, USA). For tertiary analysis, Velsera Clinical Genomics Workspace was used (Velsera). The mean depth coverage was 1000 reads. The limit of detection with high confidence was 5% mutant allele frequency with 500× minimum coverage for >95% of amplicons. Emphasis was placed on determining whether *RUNX1* variants were germline or somatic, with confirmatory testing performed on non-hematopoietic tissues (buccal swabs, hair follicles and, when required for confirmation, skin biopsy). Genetic findings were subsequently integrated with clinical and paraclinical characteristics to evaluate their association with disease presentation, clonal evolution, treatment response, and clinical outcome. Sequence variants were interpreted according to the guidelines of the Association for Molecular Pathology (AMP) and the American College of Medical Genetics and Genomics (ACMG) [61]. Additional support in variant interpretation was obtained by accessing ClinVar—Clinical Genome Resource database (https://www.clinicalgenome.org/data-sharing/clinvar/ (accessed date 15 April 2026))—and the Catalog of Somatic Mutations in Cancer (COSMIC, Wellcome Sanger Institute, Hinxton, Cambridge, UK https://cancer.sanger.ac.uk/cosmic (accessed date 15 April 2026). Variants of uncertain significance in the germline context were reported as such and were not used as the sole basis for clinical decision-making.

### 4.3. First Line Treatment and Hematopoietic Stem Cell Transplantation

Treatment strategies were individualized according to disease subtype and risk stratification. Selected patients received hypomethylating therapy with azacitidine (AZA) at a dose of 75 mg/m^2^/day for 7 consecutive days in 28-day cycles, administered as monotherapy or in combination regimens.

Allogeneic hematopoietic stem cell transplantation (HSCT) was indicated in patients with high-risk MDS or progression to acute leukemia. Donor sources included matched sibling donors (MSDs) and matched unrelated donors (MUDs). Conditioning regimens were tailored based on patient characteristics and donor type: myeloablative conditioning (MAC) using busulfan–cyclophosphamide (BuCy) or busulfan–cyclophosphamide–melphalan (BuCyMel), or reduced-toxicity conditioning (RTC) with thiotepa–treosulfan–fludarabine (ThioTreoFlu). Peripheral blood stem cells were used as graft source in all transplanted patients. Graft-versus-host disease (GvHD) prophylaxis was administered according to EBMT guidelines [62]. Preemptive donor lymphocyte infusion (DLI) was administered for mixed or declining donor chimerism, using escalating doses (1 × 10^5^/kg to >1 × 10^7^/kg CD3^+^ cells) guided by chimerism kinetics and clinical response.

### 4.4. Ethical Considerations

This study was performed in accordance with the Declaration of Helsinki. Given its retrospective, non-interventional design and the use of anonymized data, formal written informed consent for study participation was waived in accordance with institutional regulations (Ethics Committee of Fundeni Clinical Institute, approval number 3261/20.01.2026, approved on 20 January 2026). Written informed consent for genetic testing and data use was obtained from the legal guardians of all patients. BioRender was used to create the two figures, and ChatGPT v 5.4 was used for superficial text editing.

## 5. Conclusions

Overall, germline *RUNX1*-associated leukemogenesis represents a biologically distinct, evolution-driven process rather than a de novo event, with implications extending beyond conventional phenotype-based classification. This framework supports an integrated approach incorporating clonal architecture, germline context, and clinical factors while also raising the hypothesis that lineage ambiguity may represent an underrecognized manifestation of germline *RUNX1*-driven leukemogenesis in the pediatric context. Therefore, comprehensive genomic evaluation is essential for risk stratification, surveillance, and therapeutic decision-making in this setting.

## Figures and Tables

**Table 1:** Clinical and molecular characteristics of children with *RUNX1* associated with myeloid malignancies.

Patients	Case 1	Case 2	Case 3	Case 4	Case 5	Case 6
Age	4 yo	15 yo	9 yo	17 yo	6 yo	5 yo
Sex	F	F	M	M	M	M
Diagnosis	MDS/MPN	MPAL (mielo/B)	MPAL (mielo/B)	MPAL (mielo/T)	AML M7	MDS-EB
Family history	No	No	No	No	No	No
Cytogenetics/FISH	No abnormalities detected	No abnormalities detected	Complex karyotype	Complex karyotype	Monosomy 7	Monosomy 7
Gene	*RUNX1*	*RUNX1*	*RUNX1*	*RUNX1*	*RUNX1* (2 variants)	*RUNX1*
VAF (%)	43	49.1	65	41.9	18.4 9.1	29
Non-hematopoietic tissue testing	Germline	Germline	Germline	Somatic	Somatic	Somatic
Additional Tier I mutations	*NF1*	*FLT3-ITD*	*NRAS*, *CBL*	*JAK3*	None identified	*SAMD9L* (*germline*)
Additional Tier II mutations	*ALK*, *CDK4*, *APC*, *MSH2*	*EGFR* gain, *SPTA1*	*STAT3*	*MDM4*, *MYCL*, *NOTCH1*, *SMC1A*, *ATM*, *RUNX1::AFF3*, *FANCA*	None identified	*CUX1*, *BRCA2*
Firstline treatment	AZA low-dose Ara-C	CHT	CHT, AZA/VEN	CHT	CHT	AZA
Pre-HSCT MRD	Negative	0.2% (FCM)	N/A	Negative	Negative	Negative
HSCT	MUD	MUD	No	MSD	Another center	MSD
Conditioning	ThioTreoFlu	BuCyMel	Not applicable	FluThioBu	Not available	BuCy
GVHD PROPHYLAXIS	CNI, MTX, ATG	PTCy	Not applicable	CNI, MTX	Not available	CNI, MTX
GvHD grade	Acute grd IV (skin, gut)	Acute grd IV (ocular, gut)	Not applicable	No	Not available	Acute grd II (skin)
Outcome	Death (aGvHD)	Death (aGvHD)	Death (sepsis)	Alive	Post-HSCT relapse	Alive

yo, years old; mo, months; F, female; M, male; VAF, variant allele frequency; MDS, myelodysplastic syndrome; MPN, myeloproliferative neoplasm; MPAL, mixed-phenotype acute leukemia; AML, acute myeloid leukemia; Tier 1, variant of strong clinical significance; Tier II, variant of potential clinical significance; AZA, azacytidine; Ara-C, cytarabine; CHT, chemotherapy; VEN, venetoclax; MRD, minimal residual disease; FCM, flow cytometry; N/A, not applicable; HSCT, hematopoietic stem cell transplant; MUD, match unrelated donor; MSD, medular sibling donor; ThioTreoFlu, thiotepa–treosulfan–fludarabine; BuCyMel, busulfan–cyclophosphamide–melphalan; FluThioBu, fludarabine–thiotepa–busulfan; BuCy, busulfan-cyclophosphamide; CNI, calcineurin inhibitor; MTX, methotrexate; ATG, anti-thymocyte globulin; PTCy, post-transplant cyclophosphamide; grd, grade; GvHD, graft-versus-host disease; aGvHD, acute graft-versus-host disease.

**Table 2:** Molecular and functional characterization of *RUNX1* variants.

Case	Variant	Type	Exon	Functional Region	Classification	Key Evidence
**1**	c.723_729dup7 p.A244Pfs*19	frameshift	Exon 7	C-terminal region, loss of TAD	Pathogenic	LOF variant; absent controls
**2**	p.R162K c.485G>A	missense	Exon 4	RHD (DNA-binding hotspot)	Likely pathogenic	RHD hotspot; absent from controls; high deleterious computational score; public databases show classification variability, but constitutional confirmation and thrombocytopenia strongly support clinical relevance
**3**	c.227dupG p.S77Qfs*61	frameshift	Exon 3	N-terminal region, upstream of RHD (early truncation)	Pathogenic	Predicted NMD; early truncating LOF variant; absent from population databases; ClinVar expert-panel pathogenic
**4**	c.422C>A p.S141*	nonsense	Exon 4	RHD	Pathogenic	Introduces premature stop codon leading to truncation and *RUNX1* LOF; cooperative lesions for MPAL
**5**	c.352-2A>G p.unknown c.484A>G p.R162G	splice site missense	Exon 4	RHD hotspot canonical splice acceptor	Likely pathogenic (both)	Affects DNA-contact hotspot in RHD; absent from gnomAD; reported somatic in MDS/AML Disrupts canonical splice acceptor, predicted abnormal splicing and *RUNX1* LOF
**6**	c.422C>A p.S141*	nonsense	Exon 4	RHD	Pathogenic	Introduces premature stop codon leading to truncation and *RUNX1* LOF; additional somatic variants in *CUX1*, *BRCA2* and a coexisting germline SAMD9L variant; interpreted as secondary event in predisposition-like context

RHD, Runt homology domain; TAD, transactivation domain; LOF, loss of function; NMD, nonsense-mediated decay; MPAL, mixed-phenotype acute leukemia.

## Data Availability

The data presented in this study are available on request from the corresponding author due to ethical reasons.

## Abbreviations

The following abbreviations are used in this manuscript:

MDS	Myelodysplastic syndrome
AML	Acute myeloid leukemia
CBF	Core-binding factor
NGS	Next-generation sequencing
MPAL	Mixed-phenotype acute leukemia
FCM	Flow cytometry
MRD	Measurable residual disease
AMP	Association for Molecular Pathology
ACMG	American College of Medical Genetics and Genomics
AZA	Azacitidine
HSCT	Hematopoietic stem cell transplantation
MSD	Matched sibling donor
MUD	Matched unrelated donor
MAC	Myeloablative conditioning
RTC	Reduced-toxicity conditioning
GvHD	Graft-versus-host disease
EBMT	European Society for Blood and Marrow Transplantation
DLI	Donor lymphocyte infusion
DNA	Deoxyribonucleic acid
RNA	Ribonucleic acid
RHD	Runt homology domain
TAD	Transactivation domain
ID	Inhibitory domain
Hb	Hemoglobin
BM	Bone marrow
VAF	Variant allele frequency
CSA	Ciclosporine
MTX	Methotrexate
ATG	Anti-thymocyte globulin
PTCy	Post-transplant cyclophosphamide
CR	Complete remission
WBC	White blood cell count
ANC	Absolute neutrophil count
FISH	Fluorescence in situ hybridization
MDS-EB	Myelodysplastic syndrome with excess blasts

## References

[1] Locatelli F., Strahm B. (2018). How I treat myelodysplastic syndromes of childhood. Blood.

[2] Hasle H., Niemeyer C.M., Chessells J.M., Baumann I., Bennett J.M., Kerndrup G., Head D.R. (2003). A pediatric approach to the WHO classification of myelodysplastic and myeloproliferative diseases. Leukemia.

[3] Niemeyer C.M., Kratz C.P. (2008). Paediatric myelodysplastic syndromes and juvenile myelomonocytic leukaemia: Molecular classification and treatment options. Br. J. Haematol..

[4] Shimamura A., Alter B.P. (2010). Pathophysiology and management of inherited bone marrow failure syndromes. Blood Rev..

[5] Dokal I., Vulliamy T. (2010). Inherited bone marrow failure syndromes. Haematologica.

[6] Kawashima N., Oyarbide U., Cipolli M., Bezzerri V., Corey S.J. (2023). Shwachman-Diamond syndromes: Clinical, genetic, and biochemical insights from the rare variants. Haematologica.

[7] Bannon S.A., DiNardo C.D. (2016). Hereditary Predispositions to Myelodysplastic Syndrome. Int. J. Mol. Sci..

[8] Arber D.A., Orazi A., Hasserjian R.P., Borowitz M.J., Calvo K.R., Kvasnicka H.-M., Wang S.A., Bagg A., Barbui T., Branford S. (2022). International Consensus Classification of Myeloid Neoplasms and Acute Leukemias: Integrating morphologic, clinical, and genomic data. Blood.

[9] Spinner M.A., Sanchez L.A., Hsu A.P., Shaw P.A., Zerbe C.S., Calvo K.R., Arthur D.C., Gu W., Gould C.M., Brewer C.C. (2014). GATA2 deficiency: A protean disorder of hematopoiesis, lymphatics, and immunity. Blood.

[10] Marcu A.D., Bica A.M., Jercan C.G., Radu L.E., Serbanica A.N., Jardan D., Colita A., Dima S.O., Tomuleasa C., Tanase A.D. (2025). Insights into Pediatric *GATA2*-Related MDS: Unveiling Challenges in Clinical Practice. Biomedicines.

[11] Wlodarski M.W., Hirabayashi S., Pastor V., Starý J., Hasle H., Masetti R., Dworzak M., Schmugge M., van den Heuvel-Eibrink M., Ussowicz M. (2016). Prevalence, clinical characteristics, and prognosis of GATA2-related myelodysplastic syndromes in children and adolescents. Blood.

[12] Narumi S., Amano N., Ishii T., Katsumata N., Muroya K., Adachi M., Toyoshima K., Tanaka Y., Fukuzawa R., Miyako K. (2016). SAMD9 mutations cause a novel multisystem disorder, MIRAGE syndrome, and are associated with loss of chromosome 7. Nat. Genet..

[13] Tesi B., Davidsson J., Voss M., Rahikkala E., Holmes T.D., Chiang S.C.C., Komulainen-Ebrahim J., Gorcenco S., Rundberg Nilsson A., Ripperger T. (2017). Gain-of-function *SAMD9L* mutations cause a syndrome of cytopenia, immunodeficiency, MDS, and neurological symptoms. Blood.

[14] Schlegelberger B., Heller P.G. (2017). RUNX1 deficiency (familial platelet disorder with predisposition to myeloid leukemia, FPDMM). Semin. Hematol..

[15] Ripperger T., Bielack S.S., Borkhardt A., Brecht I.B., Burkhardt B., Calaminus G., Debatin K.M., Deubzer H., Dirksen U., Eckert C. (2017). Childhood cancer predisposition syndromes—A concise review and recommendations by the Cancer Predisposition Working Group of the Society for Pediatric Oncology and Hematology. Am. J. Med. Genet. Part A.

[16] Brettingham-Moore K.H., Taberlay P.C., Holloway A.F. (2015). Interplay between Transcription Factors and the Epigenome: Insight from the Role of RUNX1 in Leukemia. Front. Immunol..

[17] Cho J.Y., Akbarali Y., Zerbini L.F., Gu X., Boltax J., Wang Y., Oettgen P., Zhang D.E., Libermann T.A. (2004). Isoforms of the Ets transcription factor NERF/ELF-2 physically interact with AML1 and mediate opposing effects on AML1-mediated transcription of the B cell-specific blk gene. J. Biol. Chem..

[18] Fujimoto T., Anderson K., Jacobsen S.E., Nishikawa S.I., Nerlov C. (2007). Cdk6 blocks myeloid differentiation by interfering with Runx1 DNA binding and Runx1-C/EBPalpha interaction. EMBO J..

[19] Samarakkody A.S., Shin N.Y., Cantor A.B. (2020). Role of RUNX Family Transcription Factors in DNA Damage Response. Mol. Cells.

[20] Mangan J.K., Speck N.A. (2011). RUNX1 mutations in clonal myeloid disorders: From conventional cytogenetics to next generation sequencing, a story 40 years in the making. Crit. Rev. Oncog..

[21] Zhu F., Huang R., Li J., Liao X., Huang Y., Lai Y. (2018). Identification of Key Genes and Pathways Associated with RUNX1 Mutations in Acute Myeloid Leukemia Using Bioinformatics Analysis. Med. Sci. Monit..

[22] Brown A.L., Arts P., Carmichael C.L., Babic M., Dobbins J., Chong C.E., Schreiber A.W., Feng J., Phillips K., Wang P.P.S. (2020). RUNX1-mutated families show phenotype heterogeneity and a somatic mutation profile unique to germline predisposed AML. Blood Adv..

[23] Yamato G., Shiba N., Yoshida K., Hara Y., Shiraishi Y., Ohki K., Okubo J., Park M.J., Sotomatsu M., Arakawa H. (2018). *RUNX1* mutations in pediatric acute myeloid leukemia are associated with distinct genetic features and an inferior prognosis. Blood.

[24] He W., Zhao C., Hu H. (2020). Prognostic effect of RUNX1 mutations in myelodysplastic syndromes: A meta-analysis. Hematology.

[25] Calvo K.R., Hickstein D.D. (2023). The spectrum of GATA2 deficiency syndrome. Blood.

[26] Khan M., Cortes J., Kadia T., Naqvi K., Brandt M., Pierce S., Patel K.P., Borthakur G., Ravandi F., Konopleva M. (2017). Clinical Outcomes and Co-Occurring Mutations in Patients with RUNX1-Mutated Acute Myeloid Leukemia. Int. J. Mol. Sci..

[27] Kasahara K., Shiina M., Fukuda I., Ogata K., Nakamura H. (2017). Molecular mechanisms of cooperative binding of transcription factors Runx1-CBFbeta-Ets1 on the TCRalpha gene enhancer. PLoS ONE.

[28] Li Z., Yan J., Matheny C.J., Corpora T., Bravo J., Warren A.J., Bushweller J.H., Speck N.A. (2003). Energetic contribution of residues in the Runx1 Runt domain to DNA binding. J. Biol. Chem..

[29] Tang J.L., Hou H.A., Chen C.Y., Liu C.Y., Chou W.C., Tseng M.H., Huang C.F., Lee F.Y., Liu M.C., Yao M. (2009). AML1/RUNX1 mutations in 470 adult patients with de novo acute myeloid leukemia: Prognostic implication and interaction with other gene alterations. Blood.

[30] Bartfeld D., Shimon L., Couture G.C., Rabinovich D., Frolow F., Levanon D., Groner Y., Shakked Z. (2002). DNA recognition by the RUNX1 transcription factor is mediated by an allosteric transition in the RUNT domain and by DNA bending. Structure.

[31] Luo X., Feurstein S., Mohan S., Porter C.C., Jackson S.A., Keel S., Chicka M., Brown A.L., Kesserwan C., Agarwal A. (2019). ClinGen Myeloid Malignancy Variant Curation Expert Panel recommendations for germline RUNX1 variants. Blood Adv..

[32] Niu B., Scott A.D., Sengupta S., Bailey M.H., Batra P., Ning J., Wyczalkowski M.A., Liang W.W., Zhang Q., McLellan M.D. (2016). Protein-structure-guided discovery of functional mutations across 19 cancer types. Nat. Genet..

[33] Nurden A.T., Nurden P. (2007). Inherited thrombocytopenias. Haematologica.

[34] Cunningham L., Merguerian M., Calvo K.R., Davis J., Deuitch N.T., Dulau-Florea A., Patel N., Yu K., Sacco K., Bhattacharya S. (2023). Natural history study of patients with familial platelet disorder with associated myeloid malignancy. Blood.

[35] Natalie Deuitch M., Elizabeth Broadbridge C.G.C., Lea Cunningham B.S., Paul Liu M.D. (2021). PhD RUNX1 Familial Platelet Disorder with Associated Myeloid Malignancies. GeneReviews^®^ [Internet].

[36] Yu K., Deuitch N., Merguerian M., Cunningham L., Davis J., Bresciani E., Diemer J., Andrews E., Young A., Donovan F. (2024). Genomic landscape of patients with germline RUNX1 variants and familial platelet disorder with myeloid malignancy. Blood Adv..

[37] Ernst M.P.T., Versluis J., Valk P.J.M., Bierings M., Tamminga R.Y.J., Hooimeijer L.H., Döhner K., Gresele P., Tawana K., Langemeijer S.M.C. (2025). Disease characteristics and outcomes of acute myeloid leukemia in germline *RUNX1* deficiency (Familial Platelet Disorder with associated Myeloid Malignancy). HemaSphere.

[38] De Leon S., Sampaio De Melo M., Patel N., Dulau Florea A., Maric I., Braylan R.C., Wang W., Merguerian M., Andrews E.I., Cunningham L. (2023). Bone Marrow, Laboratory, and Clinical Features in Pediatric Patients with RUNX1 Familial Platelet Disorder with Associated Myeloid Malignancy (FPDMM). Blood.

[39] Arai H., Matsui H., Chi S., Utsu Y., Masuda S., Aotsuka N., Minami Y. (2024). Germline Variants and Characteristic Features of Hereditary Hematological Malignancy Syndrome. Int. J. Mol. Sci..

[40] Forster A., Decker M., Schlegelberger B., Ripperger T. (2022). Beyond Pathogenic RUNX1 Germline Variants: The Spectrum of Somatic Alterations in RUNX1-Familial Platelet Disorder with Predisposition to Hematologic Malignancies. Cancers.

[41] Homan C.C., Drazer M.W., Yu K., Lawrence D.M., Feng J., Arriola-Martinez L., Pozsgai M.J., McNeely K.E., Ha T., Venugopal P. (2023). Somatic mutational landscape of hereditary hematopoietic malignancies caused by germline variants in RUNX1, GATA2, and DDX41. Blood Adv..

[42] Kotmayer L., Kennedy A.L., Wlodarski M.W. (2025). Germline and somatic genetic landscape of pediatric myelodysplastic syndromes. Haematologica.

[43] Wang R.Q., Chen C.J., Jing Y., Qin J.Y., Li Y., Chen G.F., Zhou W., Li Y.H., Wang J., Li D.W. (2020). Characteristics and prognostic significance of genetic mutations in acute myeloid leukemia based on a targeted next-generation sequencing technique. Cancer Med..

[44] Sendker S., Awada A., Domagalla S., Sendker M., Orhan E., Hoffmeister L.M., Antoniou E., Niktoreh N., Reinhardt D., von Neuhoff N. (2023). RUNX1 mutation has no prognostic significance in paediatric AML: A retrospective study of the AML-BFM study group. Leukemia.

[45] Alexander T.B., Gu Z., Iacobucci I., Dickerson K., Choi J.K., Xu B., Payne-Turner D., Yoshihara H., Loh M.L., Horan J. (2018). The genetic basis and cell of origin of mixed phenotype acute leukaemia. Nature.

[46] Van der Kouwe E., Staber P.B. (2019). RUNX1-ETO: Attacking the Epigenome for Genomic Instable Leukemia. Int. J. Mol. Sci..

[47] Ptasinska A., Assi S.A., Mannari D., James S.R., Williamson D., Dunne J., Hoogenkamp M., Wu M., Care M., McNeill H. (2012). Depletion of RUNX1/ETO in t(8;21) AML cells leads to genome-wide changes in chromatin structure and transcription factor binding. Leukemia.

[48] Loke J., Assi S.A., Imperato M.R., Ptasinska A., Cauchy P., Grabovska Y., Soria N.M., Raghavan M., Delwel H.R., Cockerill P. (2017). RUNX1-ETO and RUNX1-EVI1 Differentially Reprogram the Chromatin Landscape in t(8;21) and t(3;21) AML. Cell Rep..

[49] Romanova E.I., Zubritskiy A.V., Lioznova A.V., Ogunleye A.J., Golotin V.A., Guts A.A., Lennartsson A., Demidov O.N., Medvedeva Y.A. (2022). RUNX1/CEBPA Mutation in Acute Myeloid Leukemia Promotes Hypermethylation and Indicates for Demethylation Therapy. Int. J. Mol. Sci..

[50] Li J., Jin W., Tan Y., Wang B., Wang X., Zhao M., Wang K. (2022). Distinct gene expression pattern of RUNX1 mutations coordinated by target repression and promoter hypermethylation in acute myeloid leukemia. Front. Med..

[51] Matsumura T., Nakamura-Ishizu A., Muddineni S.S.N.A., Tan D.Q., Wang C.Q., Tokunaga K., Tirado-Magallanes R., Sian S., Benoukraf T., Okuda T. (2020). Hematopoietic stem cells acquire survival advantage by loss of RUNX1 methylation identified in familial leukemia. Blood.

[52] Vannucchi A.M., Biamonte F. (2011). Epigenetics and mutations in chronic myeloproliferative neoplasms. Haematologica.

[53] Cheng C.K., Wong T.H.Y., Wan T.S.K., Wang A.Z., Chan N.P.H., Chan N.C.N., Li C.K., Ng M.H.L. (2018). RUNX1 upregulation via disruption of long-range transcriptional control by a novel t(5;21)(q13;q22) translocation in acute myeloid leukemia. Mol. Cancer.

[54] Granja J.M., Klemm S., McGinnis L.M., Kathiria A.S., Mezger A., Corces M.R., Parks B., Gars E., Liedtke M., Zheng G.X.Y. (2019). Single-cell multiomic analysis identifies regulatory programs in mixed-phenotype acute leukemia. Nat. Biotechnol..

[55] Kirtek T.J., Chen W., Harris J.C., Bagg A., Foucar K., Tam W., Orazi A., Hsi E.D., Hasserjian R.P., Wang S.A. (2026). Acute Leukemias of Ambiguous Lineage with RUNX1 Mutations Show Similar Prognosis Compared to Acute Myeloid Leukemia with RUNX1 Mutations: A Study from the Bone Marrow Pathology Group. Am. J. Hematol..

[56] Shi S., Zhou Q., Zhao D., Zarif M., Wei C., Sibai H., Chang H. (2025). Molecular genetic characterization of mixed-phenotype acute leukemia (MPAL) with BCR::ABL1 fusion. Leuk. Res..

[57] Takahashi K., Wang F., Morita K., Yan Y., Hu P., Zhao P., Zhar A.A., Wu C.J., Gumbs C., Little L. (2018). Integrative genomic analysis of adult mixed phenotype acute leukemia delineates lineage associated molecular subtypes. Nat. Commun..

[58] Bove V., Spangenberg M.N., Ottati C., Vazquez L., Catalan A.I., Grille S. (2025). Myelodysplastic syndrome with dual germline RUNX1 and DDX41 variants: A rare genetic predisposition case. Fam. Cancer.

[59] Deuitch N., Broadbridge E., Cunningham L., Liu P., Adam M.P., Bick S., Mirzaa G.M., Pagon R.A., Wallace S.E., Amemiya A. (1993). RUNX1 Familial Platelet Disorder with Associated Myeloid Malignancies.

[60] Baliakas P., Tesi B., Wartiovaara-Kautto U., Stray-Pedersen A., Friis L.S., Dybedal I., Hovland R., Jahnukainen K., Raaschou-Jensen K., Ljungman P. (2019). Nordic Guidelines for Germline Predisposition to Myeloid Neoplasms in Adults: Recommendations for Genetic Diagnosis, Clinical Management and Follow-up. HemaSphere.

[61] Richards S., Aziz N., Bale S., Bick D., Das S., Gastier-Foster J., Grody W.W., Hegde M., Lyon E., Spector E. (2015). Standards and guidelines for the interpretation of sequence variants: A joint consensus recommendation of the American College of Medical Genetics and Genomics and the Association for Molecular Pathology. Genet. Med. Off. J. Am. Coll. Med. Genet..

[62] Sureda A.C., Greco R’ Kröger S., Carreras N. (2024). The EBMT Handbook.

